# Victoria Park Hospital

**Published:** 1902-03-22

**Authors:** 


### VICTORIA PARK HOSPITAL.

### A MICROSCOPIC ATTENDANCE.

Although some 2,000 invitations had been sent out, and a notice had been inserted in half a dozen papers, besides one in Events of the Day on Tuesday morning, March 11th, only three of the governors of the City of London Hospital for Diseases of the Chest put in an appearance at Gresham College, where the annual meeting was held. The chairman, Sir Edward Sassoon, who was supported by the secretary, Mr. Dudley Ryder, eight of the committee, and Mr. Godfrey W. P. Mellor, honorary solicitor, said he was not depressed by the microscopic attendance, which was rather a proof of confidence in the methods adopted, and the general administration of the affairs of the hospital. There had been lively meetings in that room in past years, and he supposed the absence of the governors denoted that a great peace had fallen upon the meetings. The chairman then went quickly over the report, reserving detailed remarks for the occasion of the banquet at which he will preside in May, and at which he will go fully into the position of the hospital, and give some information about the scheme for the new nurses' home.

There is a very pressing need for better accommodation for the nursing staff, and, with the object of providing it, suitable plans have been selected for building a nurses' home in the grounds of the hospital at a cost of some £8,000. The urgency of this project is great, and when the representatives of King Edward's Fund inspected the hospital recently they not only approved the scheme, but recommended a grant of £100, which has since been paid.

This hospital, says the report, was the only one visited by Professor Koch during his stay in London for the Conference on Tuberculosis, and he expressed pleasure at what he saw of its work.

Both in subscriptions and in the number of patients there has been a substantial increase during the year, but legacies have been very small, and £2,000 stock has been sold to meet current expenses. Among other votes of thanks passed on Tuesday was one to the Worshipful Company of Mercers, for allowing the use of Gresham College for meetings; through their kindness a great deal of expense is saved annually. Sir Edward Sasoon, Bart., has been elected chairman of committee, in addition to the office of treasurer, which he held previously.

